# Developmental Origins and Evolution of Buchnera Host Cells

**DOI:** 10.1371/journal.pbio.0000024

**Published:** 2003-10-13

When it comes to exploiting a niche, endosymbionts take the prize. In endosymbiosis, one organism—the *endosymbiont*—invades the cells of another, in some cases taking up residence in a way that actually benefits the host. Bacteria are particularly adept at making themselves indispensable by insinuating themselves into some fundamental aspect of an organism's biology. The endosymbiotic hypothesis proposes that this is how certain eukaryotic organelles evolved from endosymbiotic bacteria. Insights into the mechanisms governing endosymbiosis will help biologists understand how this mutually beneficial relationship evolved and provide clues to one of the fundamental questions in biology: How did the eukaryotic cell evolve?

Over 10% of insect species rely on endosymbionts for their development and survival. In this issue, David Stern and colleagues look at one of the most studied pairs, the pea aphid and Buchnera aphidicola, and discover clues to the molecular foundation of their shared fate. (Buchnera, which can no longer survive outside its host cell, is thought to produce essential amino acids that the aphid cannot get on its own.)

While it is known that Buchnera are transferred from clusters of bacteriocytes in the mother to the adjacent early-stage embryo, it has been unclear how the bacteriocytes develop. Previous studies of the bacteria's genome have failed to explain the genetic basis of Buchnera's ability to invade aphid cells. Consequently, Stern and colleagues have focused on the bacteriocytes, the specialized insect cells that house Buchnera, shedding light on the development of these cells as well as on the evolutionary adaptations in the aphid that made the bacteriocytes hospitable to Buchnera.

The researchers show that bacteriocytes differentiate and proliferate independently of Buchnera's presence in the cell, and they identify three aphid transcription factors (proteins that regulate gene expression) that are expressed in three distinct stages during early-bacteriocyte development in the aphid embryo. The first protein is expressed just before Buchnera enters the embryo; a second, as the bacteria invades; and a third, after the transfer is nearly complete. A second wave of the same transcription factors occurs at a later stage in aphid embryo development and increases the population of bacteriocytes.

This two-step specification of bacteriocytes, which occurs in related Buchnera-carrying aphid species, appears to be an evolutionarily conserved feature of aphids. It even occurs in an aphid species that once had a Buchnera endosymbiont and now has a yeast-like symbiont that lives outside the bacteriocytes. But this process is not observed in males of another aphid species that do not carry Buchnera. While traces of the first transcription factor activated in bacteriocytes are evident, the characteristic gene-expression pattern is not, and the aphids have no mature bacteriocytes.

While it seems that the aphid has evolved new domains of expression in the bacteriocyte for these transcription factors—none of these transcription factors is expressed at a similar stage in other insects—the researchers cannot yet say whether these genes direct the specification of bacteriocytes. Still, these transcription factors are likely to play important roles in the bacteriocyte, suggesting that the union of aphids and Buchnera involved significant adaptations by the host.

**Figure:**
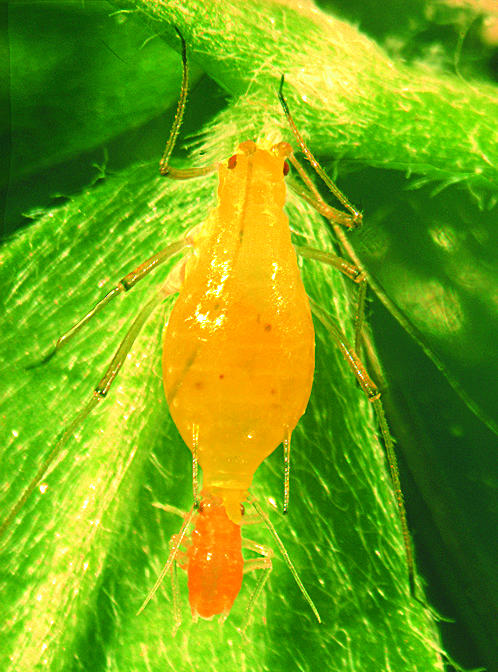
Aphid host of Buchnera endosymbionts

